# Histopathologic Characterization and Neurodegenerative Markers in Patients With Limbic Encephalitis Undergoing Epilepsy Surgery

**DOI:** 10.3389/fneur.2022.859868

**Published:** 2022-04-15

**Authors:** Attila Rácz, Chiara A. Hummel, Albert Becker, Christoph Helmstaedter, Fabiane Schuch, Tobias Baumgartner, Randi von Wrede, Valeri Borger, László Solymosi, Rainer Surges, Christian E. Elger

**Affiliations:** ^1^Department of Epileptology, University Hospital Bonn, Bonn, Germany; ^2^Department of Neuropathology, University Hospital Bonn, Bonn, Germany; ^3^Department of Neurosurgery, University Hospital Bonn, Bonn, Germany; ^4^Department of Neuroradiology, University Hospital Bonn, Bonn, Germany

**Keywords:** limbic encephalitis, hippocampal sclerosis, neurodegenerative markers, epilepsy surgery, GAD65 antibodies

## Abstract

**Purpose:**

Limbic encephalitis is an increasingly recognized cause of medial temporal lobe epilepsy (mTLE) and associated cognitive deficits, potentially resulting in hippocampal sclerosis (HS). For several reasons, these patients usually do not undergo epilepsy surgery. Thus, histopathologic examinations in surgical specimens of clearly diagnosed limbic encephalitis are scarce. The purpose of this study was a detailed histopathologic analysis of surgical tissue alterations, including neurodegenerative markers, in patients with limbic encephalitis undergoing epilepsy surgery.

**Methods:**

We investigated the surgical specimens of six patients operated on with mTLE related to limbic encephalitis (among them four patients were with GAD65 and one with Ma1/2 antibodies), and compared the findings to a control group with six patients matched according to age at the time of surgery without limbic encephalitis and without early inciting events.

**Results:**

Histopathologic analysis in the group with limbic encephalitis revealed HS in four patients, while three of them also displayed signs of an active inflammatory reaction with lymphocytes. In one of the patients with GAD65-encephalitis who was suffering from a late-onset mTLE and a long disease course, neurodegenerative protein markers (β-amyloid and hyperphosphorylated tau) were found coexisting with inflammatory reactions and HS. Investigations in the control group did not reveal any inflammatory reaction or neurodegenerative marker.

**Conclusion:**

Our findings suggest a possible link between long-lasting immune reactions in the medial temporal lobe, HS, and further toward the development of neurodegenerative diseases. Presently, however, a causal relationship between these entities cannot yet be established. Furthermore, our results suggest that an immunological etiology should always be considered in late onset (> 18 years) mTLE, also in cases of long disease duration and the presence of HS.

## Introduction

A large percentage of focal epilepsies originate in the medial temporal lobe, and epilepsy surgery often renders these patients seizure-free (1). Recent developments in neuroimmunology and magnetic resonance imaging (MRI) have revealed that a substantial number of temporal lobe epilepsies has an immunological/inflammatory origin, leading to the diagnostic concept of limbic encephalitis, illnesses typically associated with temporomesial T2/FLAIR-hyperintensities and amygdala enlargement in MRI images (2–8). Although in many cases limbic encephalitis is associated with specific antibodies (3, 7, 8), in several cases the search for antibodies remains fruitless, and a so-called seronegative limbic encephalitis is postulated (6).

Limbic encephalitis often results in HS; however, only a few series of patients undergoing epilepsy surgery with limbic encephalitis have been reported so far (9–11). Possible reasons for this bias can be attributed to the frequent bilateral affection with multiple seizure generators hampering the possibility of curative epilepsy surgery, as well as uncertainties regarding potential re-inflammation after epilepsy surgery or during the later course of the disease. The reported series are of small sample size; nonetheless, they reflect that different antibodies are possibly associated with different postsurgical outcomes, glycine receptor antibodies, for instance, with rather favorable (9), and GAD65 antibodies with rather unfavorable outcomes (11, 12).

An overlap between histopathological and biochemical substrates of HS (though primarily in elderly, non-epileptic patients), dementia, and Alzheimer's disease has also long been speculated in the literature, and HS has been shown to be associated with an increased risk for dementia (13–15). In addition, epilepsy is supposed to contribute to neurodegenerative brain tissue alterations, and a substantial proportion of patients with temporal lobe epilepsy between 50 and 65 years of age were shown to exhibit an increased amount of neurodegenerative protein markers in surgical specimens (16, 17).

Our goal was to precisely characterize histopathologic findings in a special group of patients with medically intractable mTLE due to limbic encephalitis (four patients with GAD65 antibodies and one with Ma1/2 antibodies) compared to a control group matched to the first group according to age at the time of surgery. In particular, we focused on the presence or absence of possible inflammatory reactions and signs of neurodegenerative protein markers in the resected specimens.

## Patients and Methods

Six patients suffering from medically difficult-to-treat mTLE within the context of limbic encephalitis and an additional six patients with medically difficult-to-treat temporal lobe epilepsy without limbic encephalitis and without early inciting events, matched according to age at the time of surgery, and operated on between 2002 and 2020 in our center were selected from our electronic database. The detailed characteristics of these patients (including potential psychiatric aspects during the disease course) are outlined in Table 1 (limbic encephalitis group) and Table 2 (control group). All these patients underwent a comprehensive presurgical diagnostic workup comprised of a brain MRI, video-EEG examinations, and neuropsychological evaluation. Histopathologic analysis of surgical specimens from these patients, including examinations of neurodegenerative protein markers, was performed in the Department of Neuropathology at the University Hospital Bonn. For three patients in the limbic encephalitis group and five patients in the control group, invasive presurgical diagnostics preceded resective epilepsy surgery. In patients undergoing invasive monitoring in the limbic encephalitis group, bitemporal subdural electrodes (three patients) and also hippocampal depth electrodes on one side (one patient) or both sides (two patients) were applied. In each case, a unilateral seizure generator could be verified. In patients undergoing invasive diagnostics in the control group bitemporal (including hippocampal) depth electrodes (five patients), and in one patient additional subdural electrodes were used, and a unilateral seizure generator could be verified in two patients. In the limbic encephalitis group four patients had GAD65 antibodies, one patient had Ma1/Ma2 antibodies (in the context of a paraneoplastic limbic encephalitis), and another patient displayed voltage-gated potassium channel (VGKC) antibodies. Since in this last patient neither LGI1 nor CASPR2 antibodies could be detected in serologic tests performed later, we designated him seronegative later on. In three patients, limbic encephalitis was diagnosed preoperatively, while in the remaining three patients from the limbic encephalitis group during the postoperative course (though in one of these patients limbic encephalitis was hypothesized already before surgery). In four patients, a diagnosis of HS was made based on MRI already before surgery (also correlating with histopathologic diagnosis after surgery), and in one of them, a bilateral HS was present. In this particular case, limbic encephalitis with severe cognitive impairment was diagnosed on the contralateral side 27 months after epilepsy surgery and it responded well to steroids (Table 1). Though this patient presented a long epilepsy duration and bilateral HS before surgery, the later clinical course makes it probable that the primary reason for mTLE in his case was limbic encephalitis. In four of the six patients in the limbic encephalitis group, we could definitely identify a subacute onset (or at least an onset within a short time period) with temporomesial signal alterations in brain MRI, new onset temporal lobe seizures, and/or memory deficits typical of limbic encephalitis. For the remaining two patients, we were unable to trace back the complete case history, but in these patients antibody tests and histopathologic findings contributed to the diagnosis. Antibody screening was also performed in four patients from the control group: in three patients in serum as well as in cerebrospinal fluid (CSF); in one patient in serum only, yielding no specific finding. In one of these patients, limbic encephalitis was hypothesized for the severe cognitive deficits; nevertheless, serologic tests in serum and CSF were unrewarding, and histopathologic examinations in the surgical specimen did not show inflammatory changes either. Certain features of some of these patients and some aspects of histopathologic findings have been described and published elsewhere (10, 12). Postsurgical outcomes regarding seizure control were scaled according to the Engel-classification (18). As a standard, epilepsy surgical patients sign a consent for scientific investigations on surgical specimens before surgery. The study was approved by the ethics committee of the Medical Faculty of the University of Bonn (Nr. 360/12).

**Table 1:** Clinical characteristics of patients undergoing temporal lobe surgery with limbic encephalitis.

**Antibody**	**Gender**	**Tumor**	**Age at epilepsy onset**	**Age at surgery**	**Side/surgery**	**MRT diagn. HS**	**Psychiatric features**	**LE diag**.	**Immunotherapy**	**Histology (HS)**	**Histology (inflammation)**	**Histology (neurodeg)**	**Outcome at longest FU**
GAD65	Female	No	24,4	26,5	Left (sAHE)	No	Depressed mood postop, emotional instability	Preop	Steroids, IA, liquor drainage, cyclophosphamide, mycophenolate mofetil	No	Yes	No	II (110 months)
GAD65	Male	No	25	35,1	Left (sAHE)	No	Aggression	Postop	Azathioprine	No definite	Yes	No	II (121 months)
*GAD65*	Female	No	23	23,7	Right (sAHE)	Yes	Depressed mood	Postop	Steroids, IVIG, azathioprine, IA	Yes	Yes	No	III (83 months)
GAD65	Female	No	30,6	53,6	Left (sAHE)	Yes	PNES, aggression, depressed mood	Preop	Steroids	Yes	Yes	Yes	III (11 months)
Ma1/Ma2	Female	Yes	15	20,1	Left (ant temp lobectomy)^*^	Yes	Depression, anxiety	Preop	Steroids, mycophenolate mofetil	Yes	Yes	No	IA (48 months)^**^
Seroneg^***^	Male	No	8	33,1	Left (sAHE)	Yes^****^	Irritability and suicide ideation under steroids	Postop	Steroids (27 months after surgery)	Yes	No	No	IA (36 months)

* *Two surgeries*.

** *Outcome after the second surgery (roughly 7 months after first surgery)*.

*** *VGKC (no LGI1, no CASPR2) and low titer NMDAR IgM antibodies (unspecific finding)*.

**** *On both sides. FU, follow-up; IA, immunadsorption; IVIG, intravenous immunglobuline; LE, limbic encephalitis; HS, hippocampal sclerosis; PNES, psychogenic, non-epileptic seizures; sAHE, selective amygdalohippocampectomy. Patients with antibodies in italic script presented a contralateral inflammation more than 1 year after surgery*.

**Table 2:** Clinical characteristics of patients undergoing temporal lobe surgery without limbic encephalitis and without an early inciting event (control group).

**Gender**	**Age at epilepsy onset**	**Age at surgery**	**Side/surgery**	**MRT diagn. HS**	**Psychiatric features**	**Histology (HS)**	**Histology (inflammation)**	**Histology (neurodeg)**	**Outcome at longest FU**
Female	5,5	20,5	Left (sAHE)	Yes	Depression, anxiety, impulsivity, postictal psychosis	Yes	No	No	II (24 months)
Female	13	24,1	Left (sAHE)	Yes	Depression	Yes	No	No	II (34 months)
Male	14	26,9	Right (sAHE)	No	Depression (probably related to AED treatment)	Yes	No	No	IA (12 months)
Male	19	33,5	Left (sAHE)	Yes (right) / possibly (left)	Depressed mood, irritability after seizures	No	No	No	III (54 months)
Male	18	34,8	Left (sAHE)	Yes	Anxiety (probably related to AED treatment), postictal aggression	Yes	No	No	IA (14 months)
Male	46	52,9	Left (sAHE)	Yes	Depression	Yes	No	No	IA (49 months)

*HS, hippocampal sclerosis; FU, follow-up; sAHE, selective amygdalohippocampectomy; AED, antiepileptic drug*.

## Results

Histopathologic alterations were examined in six patients with pharmacologically difficult-to-treat mTLE and associated limbic encephalitis and another six patients without limbic encephalitis, and they were matched according to age at the time of surgery. Detailed characteristics of these patients are outlined in Tables 1, 2. Epilepsy surgery took place at 32.02 ± 4.90 years in the limbic encephalitis group and 32.12 ± 4.72 years in the control group (mean ± standard error of the mean). Although it was not possible to further match patients according to age at epilepsy onset and duration of epilepsy, the groups presented comparable values in these respects as well (age at onset: 21 ± 3.31 years for the limbic encephalitis group and 19.26 ± 5.69 years for the control group; duration of epilepsy: 11.02 ± 4.34 years for the limbic encephalitis group and 12.87 ± 1.42 years for the control group). In the limbic encephalitis group, histopathologic examinations revealed HS in four patients (with or without inflammatory cell reactions) and lymphocytic infiltrations without definite signs of hippocampal sclerosis in the remaining two patients (in one of these patients, however, histopathologic findings were suspicious for HS). In the patient with Ma1/2 antibodies, two surgeries were performed (anterior temporal lobectomy and an extension of the temporal lobectomy) as the first surgery did not result in seizure freedom. Interestingly, histopathologic investigations in three patients revealed HS and also inflammatory cell reactions reconcilable with active encephalitis (Tables 1, 3).

**Table 3:** Neuropathologic findings in patients undergoing temporal lobe surgery with limbic encephalitis and in the control group.

**LE group – Age at surgery (years)**	**26,5**	35,1^*^	23,7^**^	**53,6**	20,1^***^	**33,1**
Antibody	GAD65	GAD65	GAD65	GAD65	Ma1/Ma2	Seroneg. (VGKC)
HS – Wyler grade	No def. HS	Susp. of Wyler II	Not further class.	Wyler II	Wyler III	Wyler IV
HS - ILAE	No def. HS	Susp. of ILAE 3	ILAE 1	ILAE 3	ILAE 1	ILAE 1
Astrogliosis	Yes	Yes	Yes	Yes	Yes	Yes
Microglial activation	Yes	Yes	Yes	Yes	Yes	Yes
Lymphocytes	CD8 (> CD20)	CD3, esp. CD8	CD3, CD20	CD3, esp. CD8 (>CD20)	Not further class.	No
**Control group –** **Age at surgery (years)**	26,9^****^	34,8^*****^	**24,1**	**52,9**	**20,5**	**33,5**
HS – Wyler grade	Not further class.	Not further class.	Wyler IV	Wyler III	Wyler IV	No HS
HS - ILAE	Not further class.	ILAE 1	ILAE 1	ILAE 1	ILAE 1	No HS
Astrogliosis	Yes	Yes	Yes	Yes	Yes	Yes
Microglial activation	Yes	Yes	Not classifiable	Yes	Yes	Yes
Lymphocytes	No	No	No	No	No	No

* *In this particular patient no definite histopathological diagnosis was possible (fragmented tissue), however, tissue changes were suspicious for an “end folium” sclerosis*.

** *No definite classification according to the Wyler system possible, fragmented tissue*.

*** *In this patient brain biopsy performed early in the course of disease in another hospital showed already T-cell mediated encephalitis*.

**** *Hippocampal subregions could not be clearly identified*.

***** *No definite classification according to the Wyler system possible. LE, limbic encephalitis; ILAE, International League Against Epilepsy; HS, hippocampal sclerosis*.

**Figure 1:**
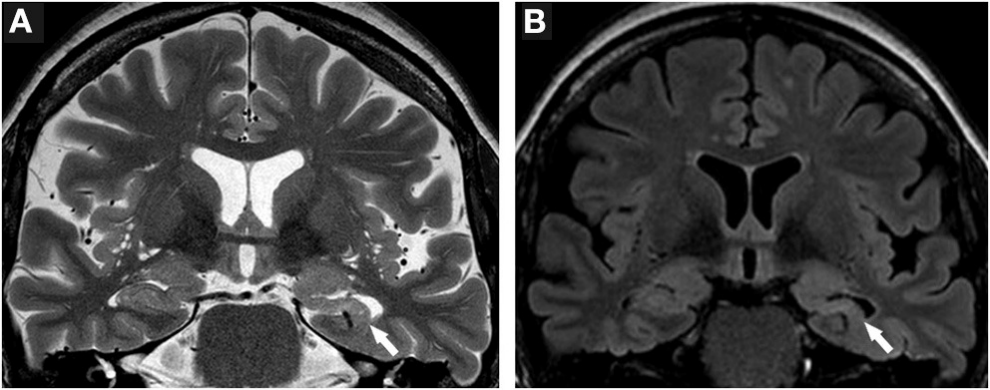
Neuroimaging findings in Patient #4. **(A)** T2-weighted brain MRI scans reveal hippocampal volume loss and a markedly disturbed inner structure of the hippocampus on the left side (white arrow). **(B)** FLAIR images further highlight volume loss and signal hyperintensity of the left hippocampus (white arrow). Structures of the right medial temporal lobe do not show pathologic signal alterations.

**Figure 2:**
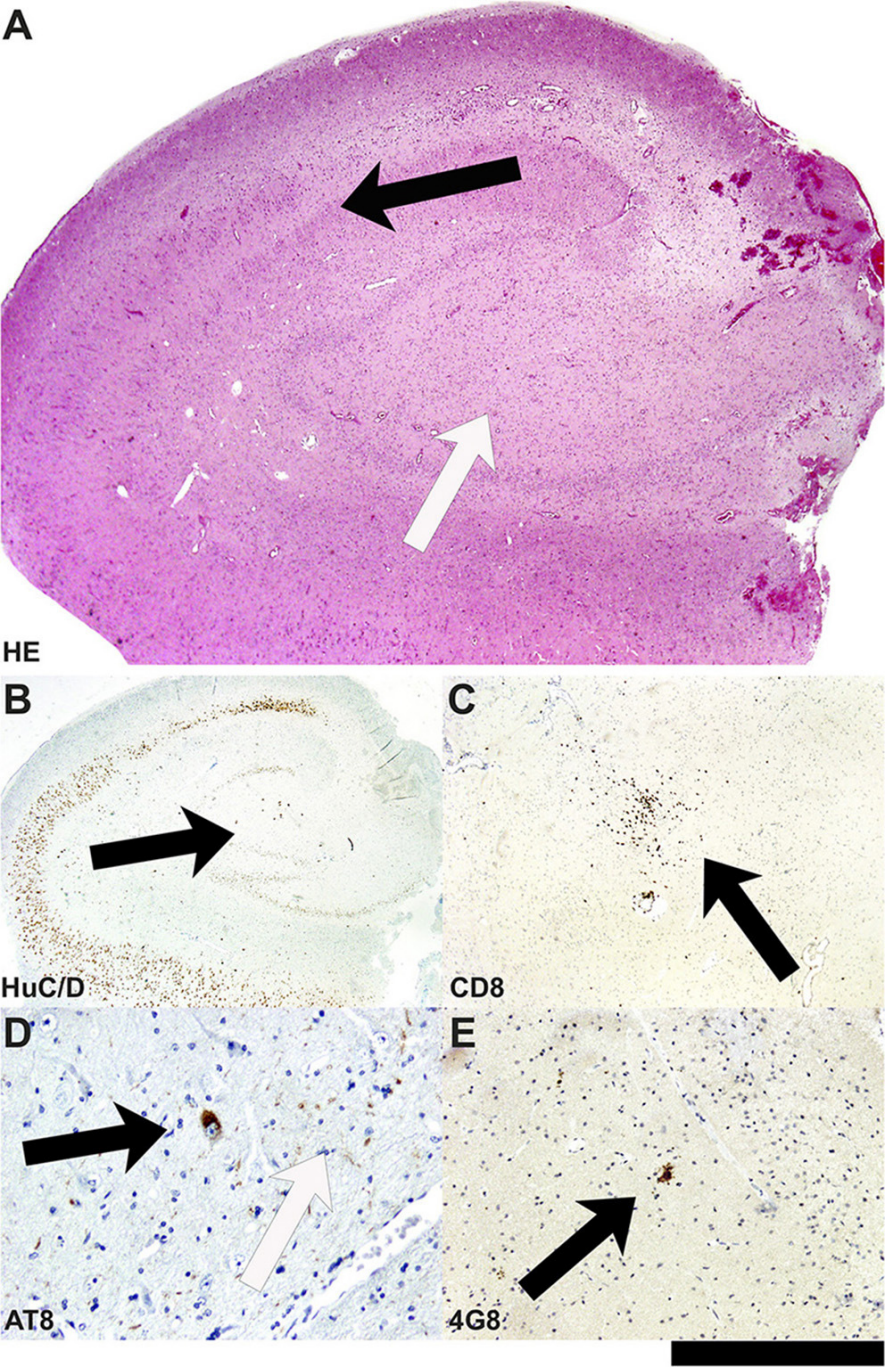
Neuropathological findings in the hippocampal biopsy specimen in Patient #4. **(A)** On the HE-stained section, the hippocampal formation reveals segmental neuronal cell loss in the hilus/CA4 as well as CA3 area (white arrow), whereas the neuronal population in the CA1 area and others is rather well conserved (black arrow). **(B)** Segmental neuronal cell loss in hilus/CA4 is also highlighted in immunohistochemistry with antibodies against HuC/D (black arrow). **(C)** In the hilus/CA4 region, clusters of CD8-positive cytotoxic lymphocytes are present (black arrow). **(D)** Surprisingly, not only extracellular AT-8 positive tau fibrils (white arrow) but also intracellular tangle-like tau structures can be seen in neurons of this hippocampal formation (black arrow). **(E)** In parallel, 4G8-positive amyloid-plaque structures represent parallel extracellular deposits (black arrow; bar graph corresponds to 1.0 mm in A, 2.0 mm in B, 0.5 mm in C; 100 μm in D, 200 μm in E).

**Figure 3:**
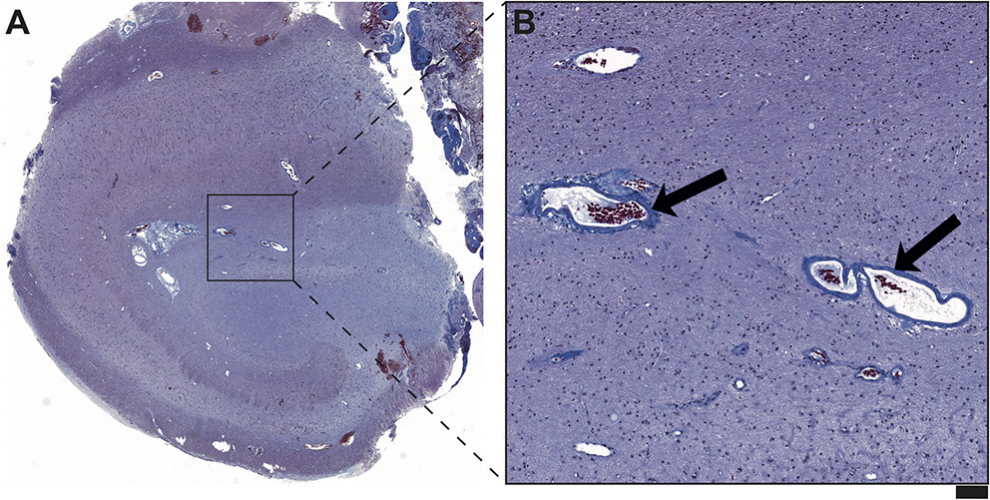
**(A)** On the Masson Trichrom staining, no signs of microvessel disease or fibrinoid necrosis in the hippocampal formation of Patient #4 are detected. **(B)** The figure reveals representative vessels with regular wall structure (arrows) in the hippocampal area with maximal neuronal damage. No significant vascular wall inflammatory infiltrates are visible. Overall, there also were no neuropathological correlates of vasculitis in this hippocampal formation. Scale bar corresponds to 500 μm in A and to 100 μm in B.

Moreover, the disease course for one of these patients (Patient #4) with late-onset mTLE (30 years) was very long, amounting to 23 years. MRI diagnostics before surgery revealed HS on the left side and no abnormalities on the right side (Figures 1A,B). The patient also suffered from diabetes mellitus and hypothyroidism (elevated TPO antibodies). The non-invasive presurgical workup was concordant in determining that the HS was the seizure generator (semiology, interictal, and ictal EEG alluding to the left temporal region; neuropsychological investigations revealing global cognitive impairment, but most pronounced left temporal-lobe-related verbal memory deficits). Cerebellar atrophy could not be visualized with MRI; nevertheless, the patient also displayed a certain supratentorial brain atrophy on MRI images. Limbic encephalitis was diagnosed before surgery. Due to late-onset mTLE and the absence of classical precipitating events (e. g., febrile seizures), a lumbar puncture and antibody diagnostics were performed and revealed a high titer (1:3200) of GAD65-antibodies in the serum and a moderate, but still significant, GAD65-antibody titer (1:32) in the CSF, as well as a slightly elevated cell count (7/μl) in CSF and intrathecal IgG-synthesis. After surgery, for which the chance of seizure freedom was estimated to be roughly 50%, histopathologic examinations confirmed HS, predominantly affecting the CA4 region corresponding to HS ILAE Type 3 and Wyler grade II [(19, 20); Figures 2A,B]. Surprisingly, other than T-cell-dominated inflammatory reactions (clusters of CD8-positive cytotoxic lymphocytes, Figure 2C), further stainings on the resected specimen also revealed hyperphosphorylated tau and β-amyloid plaques, indicating a possible neurodegenerative process (Figures 2D,E). To further evaluate whether neurodegenerative changes in this patient might have come about as a result of potential vascular changes related to diabetes mellitus or as a result of a vasculitis (potentially related to an autoimmune thyroid disease), we performed a Masson-Trichrom staining. Neuropathologically, the blood vessel structures appeared intact without definite alterations resembling microangiopathy, either in the hematoxylin-eosin or the Masson-Trichrom staining (Figure 3). The patient displayed further deterioration in neuropsychological tests after surgery, and one might hypothesize that the global deficits in her case could signalize a further development toward dementia.

For the remaining five patients, neurodegenerative protein markers could not be verified on histopathological sections (Tables 1, 3). Altogether, none of the four patients with GAD65-encephalitis became seizure-free after surgery, whereas the patient with Ma1/2 antibody was seizure-free after the second surgery, and the seronegative patient also attained complete seizure freedom (Table 1).

**Figure 4:**
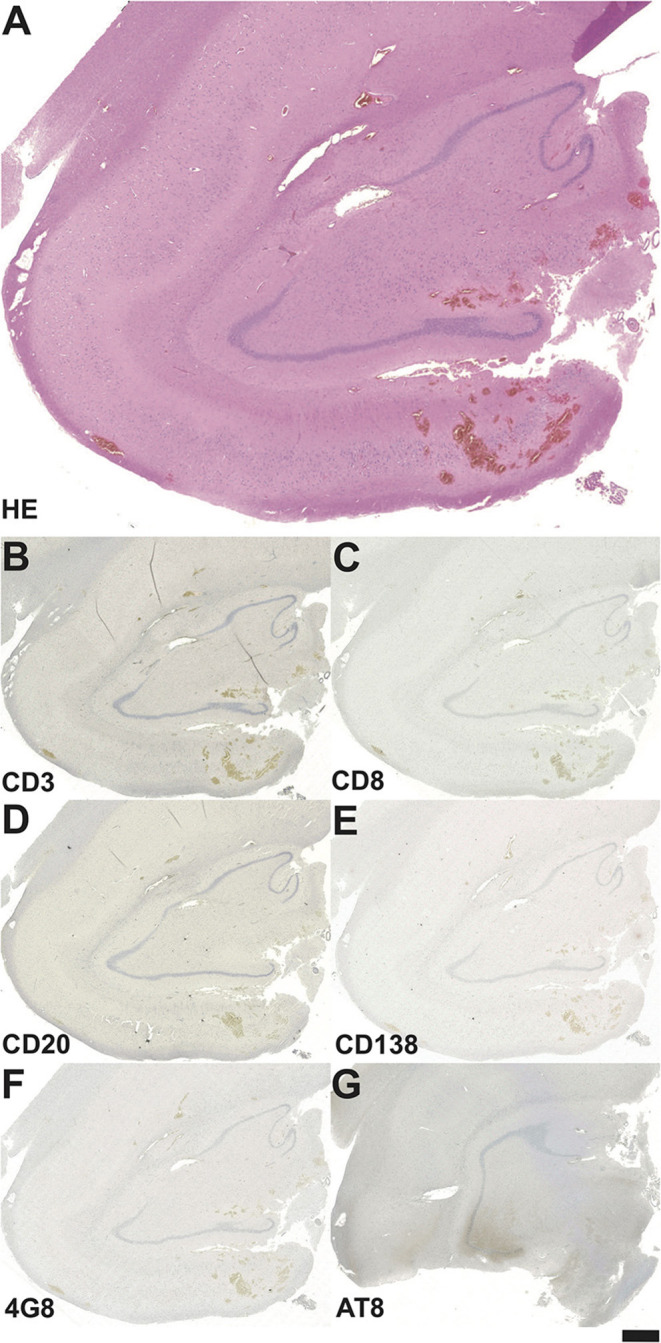
Neuropathological findings in the hippocampal biopsy specimen in a patient from the control group operated on at the age of 33 years. **(A)** On the HE-stained section no definite signs of HS can be seen. Staining against the T-cell marker CD3 **(B)**, cytotoxic T-lymphocyte marker CD8 **(C)**, the B-cell marker CD20 **(D)** and the plasma cell marker CD138 **(E)** do not reveal any inflammatory cell reaction. **(F)** Among neurodegenerative markers, there is no hint of 4G8-positive amyloid plaques, **(G)** AT-8 positive tau fibrils are missing as well. Bar graph corresponds to 500 μm in A, and 1.0 mm in B-G.

In five out of the six patients in the control group, HS could be verified in histopathology (Tables 2, 3). None of these patients exhibited signs of inflammatory lymphocytic reactions or neurodegenerative protein markers (Tables 2, 3 and Figure 4). Three patients achieved complete seizure freedom, while two patients displayed an Engel class II outcome, and another patient had an Engel class III outcome after epilepsy surgery (Table 2).

**Figure 5:**
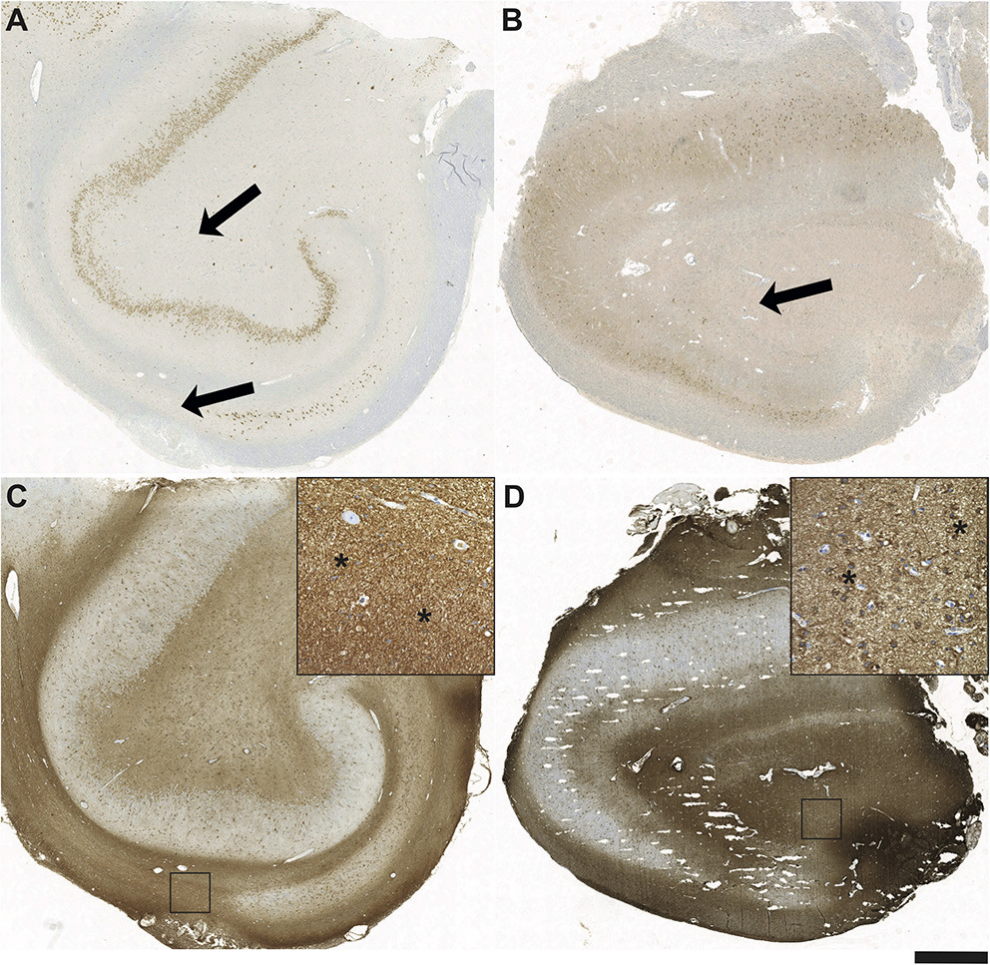
Fibrillary and cellular astrogliosis in antibody positive HS Type 3 and seronegative HS Type 1. Sections after immunohistochemistry with antibodies against NeuN demonstrating a hippocampal formation of **(A)** a seronegative patient with pharmacorefractory TLE and HS Type 1 with subtotal segmental neuronal loss in CA1 (arrows) and also in CA3/CA4 in contrast to **(B)** the patient positive for GAD65 antibodies with “*end folium sclerosis”* given by subtotal neuronal loss in the hilus (arrow; HS Type 3). Note also the severe neurodegeneration of dentate gyrus (DG) granule cells. Glial fibrillary acidic protein (GFAP) positive astrogliosis is most strongly present in hippocampal areas with the most massive neuronal loss in CA1 in Type 1 HS **(C)** vs. CA3/CA4 in Type 3 HS **(D)**. Note the different types of mainly fibrillary gliosis in Type 1 (insert in **C** – magnification from square; asterisks) vs. pronounced cellular astrogliosis in Type 3 HS (insert in **D** – magnification from square; asterisks); scale bar corresponds to 1,000 μm in A-D and to 100 μm in inserts.

A more detailed overview of the histopathologic findings (including astrogliosis, microglial activation, and infiltrating lymphocytes) is given in Table 3. Interestingly, in the control group, surgical specimens with HS (if classifiable) exclusively displayed ILAE Type 1 HS, whereas in the limbic encephalitis group one patient (Patient #4) had HS ILAE Type 3 (previously designated as “end folium sclerosis”), and findings in another patient were suspicious for HS ILAE 3 (suspicious but not definite due to the fragmented biopsy condition; Table 3). In many cases, lymphocyte infiltrations in the limbic encephalitis group were characterized by a predominance of CD8 cells (Table 3). Distinct aspects of astrogliosis are shown in Figure 5. In a patient without limbic encephalitis and with HS ILAE Type 1, we see mainly fibrillary astrogliosis (Figures 5A,C), whereas in a patient with GAD65 antibodies and HS Type 3, a more pronounced cellular astrogliosis is present (Figures 5B,D).

## Discussion

In this study, we present and discuss histopathologic findings in six patients with mTLE who were operated on within the context of limbic encephalitis and compare them against a matched control group according to age at the time of surgery. Interestingly, half of the patients with limbic encephalitis displayed HS as well as an inflammatory cell reaction in the surgical histopathological specimen; in two patients even several years after disease onset. Moreover, in the resected hippocampus of Patient #4 with late-onset mTLE (30 years of age) and GAD65-encephalitis, neurodegenerative protein markers could also be identified. GAD65 antibodies are associated with a variable spectrum of disorders (21–25). Limbic encephalitis with GAD65-antibodies is known to be a chronic and long-lasting autoimmune encephalitis with frequent failure of immunomodulatory and anticonvulsive treatments (12, 26–29). In addition to suffering from epilepsy, patients usually display cognitive deficits (28–30). This particular case highlights novel aspects of limbic encephalitis and a possible, yet hypothetical link to neurodegenerative diseases. First, even though cases with histologically-verified HS and associated limbic encephalitis have already been reported (11), to our knowledge, a disease duration of this length (overall 23 years) with histopathologic evidence of an active encephalitis coexistent with HS has not yet been observed. Moreover, the presence of protein aggregates specific for a neurodegenerative process, β-amyloid and hyperphosphorylated tau, provide a possible link between long-lasting inflammatory processes in the temporal lobe and the development of neurodegenerative diseases, especially Alzheimer's disease. Previous studies have shown that temporal lobe epilepsy may result in neurodegenerative alterations (16, 17); as potential explanatory mechanisms for this phenomenon, seizure-related trauma and pathological epileptic activity have been discussed (17). Based on our findings, a further possible mechanism can be postulated: in the discussed histopathological specimen from Patient #4, a complete spectrum of a most likely inciting T-cell mediated inflammatory reaction (31), as a result of that an HS, and as a further possible, currently, yet speculative step, neurodegenerative protein aggregates could be confirmed.

The presence of T-lymphocytes is not specific to immune-mediated mTLE. It has also been shown in surgical specimens of primarily non-immune mediated mTLE (32) and also in Alzheimer's disease (32, 33). In Alzheimer's disease, T-cell presence seems to be a secondary effect induced by neurodegenerative mechanisms (33). In distinct types of mTLE, there appears to be a correlation between the number of T-cells and the degree of neuronal loss; however, the direction of causality between the two variables is unclear so far (32). In our control series, T-cells were not detectable; however, the spectrum of mTLE etiologies in the cited study (32) was different from our control series, since we did not include patients with early inciting events such as febrile seizures or patients with glioneuronal tumors.

Neurodegenerative markers could not be identified in the remaining five patients from the limbic encephalitis group nor in any of the patients from the control group. Surgery in the remaining patients with limbic encephalitis took place at the age of 26, 35, 23, 20, and 33 years respectively (Table 1); and duration of epilepsy was 2, 10, 1 (8 months), 5, and 25 years. In fact, we cannot completely exclude the possibility that given the abundance of Alzheimer's pathology in the current population, the co-occurrence of HS and neurodegenerative protein markers in Patient #4 is merely a coincidence. On the other hand, relevant neurodegenerative alterations were reported to be mainly found in the age spectrum of 50–65 years for patients with epilepsy (16). From our series, only Patient #4 fell in this age group at the time of surgery. However, the duration of epilepsy and presumably that of the encephalitic process was, with one exception, reasonably shorter for the remaining patients compared to Patient #4. Therefore, one might speculate that a hypothetical “evolution” to a neurodegenerative stage for patients with limbic encephalitis needs a reasonably long time course, and one may not have the chance to see it after only a few years.

Our study has certain limitations. The observation on the association of inflammatory cell reaction, HS, and neurodegeneration is based on one case, and there is no firm evidence for a causal relationship between limbic encephalitis and neurodegenerative alterations. The observation that limbic encephalitis might be more frequently associated with HS ILAE Type 3 is interesting; however, due to the low number of cases, the relevance of this finding is unclear to date. These aspects and the described hypothesis should be investigated on a larger group of patients and possibly in a multicenter setting.

The observations in the presented series also have important clinical implications. In the seronegative patient (VGKC antibodies), limbic encephalitis was diagnosed on the contralateral side 27 months after surgery. In the patient with GAD65-antibodies and neurodegenerative protein markers (Patient #4), the results of the presurgical diagnostic workup were fairly concordant and underpinning a unilateral involvement. Further follow-ups, however, revealed aspects alluding to the affection of both medial temporal lobes. Thus, in patients with late-onset temporal lobe epilepsy and one-sided HS, a possible autoimmune inflammatory origin and a “hidden” bitemporal affection should always be considered, even if the presurgical workup shows otherwise concordant results.

## Data Availability Statement

The raw data supporting the conclusions of this article will be made available by the authors, without undue reservation.

## Ethics Statement

The studies involving human participants were reviewed and approved by Ethikkommission der Medizinischen Fakultät der Rheinischen Friedrich-Wilhelms-Universität Bonn. The patients/participants provided their written informed consent to participate in this study.

## Author Contributions

AR, AB, RS, and CEE contributed to conception and design of the study. AR, CAH, AB, CH, FS, TB, RW, VB, and LS collected the data. AR, CAH, and AB analyzed the data. AR, AB, and CEE wrote the first draft of the manuscript. All authors contributed to manuscript revision, read, and approved the submitted version.

## Funding

AR is employed by the Department of Epileptology, University Hospital Bonn. CAH's MD thesis work was supported by a grant-in-aid from the Else Kröner-Fresenius-Foundation.

## Conflict of Interest

The authors declare that the research was conducted in the absence of any commercial or financial relationships that could be construed as a potential conflict of interest.

## Publisher's Note

All claims expressed in this article are solely those of the authors and do not necessarily represent those of their affiliated organizations, or those of the publisher, the editors and the reviewers. Any product that may be evaluated in this article, or claim that may be made by its manufacturer, is not guaranteed or endorsed by the publisher.
